# Gene Therapy in a Humanized Mouse Model of Familial Hypercholesterolemia Leads to Marked Regression of Atherosclerosis

**DOI:** 10.1371/journal.pone.0013424

**Published:** 2010-10-19

**Authors:** Sadik H. Kassim, Hui Li, Luk H. Vandenberghe, Christian Hinderer, Peter Bell, Dawn Marchadier, Aisha Wilson, Debra Cromley, Valeska Redon, Hongwei Yu, James M. Wilson, Daniel J. Rader

**Affiliations:** 1 Gene Therapy Program, Department of Pathology and Laboratory Medicine, University of Pennsylvania, Philadelphia, Pennsylvania, United States of America; 2 Institute for Translational Medicine and Therapeutics, University of Pennsylvania, Philadelphia, Pennsylvania, United States of America; Maastricht University, Netherlands

## Abstract

**Background:**

Familial hypercholesterolemia (FH) is an autosomal codominant disorder caused by mutations in the low-density lipoprotein receptor (*LDLR*) gene. Homozygous FH patients (hoFH) have severe hypercholesterolemia leading to life threatening atherosclerosis in childhood and adolescence. Mice with germ line interruptions in the *Ldlr and Apobec1* genes (*Ldlr^−/−^Apobec1^−/−^*) simulate metabolic and clinical aspects of hoFH, including atherogenesis on a chow diet.

**Methods/Principal Findings:**

In this study, vectors based on adeno-associated virus 8 (AAV8) were used to deliver the gene for mouse *Ldlr* (*mLDLR*) to the livers of *Ldlr^−/−^Apobec1^−/−^* mice. A single intravenous injection of AAV8.*mLDLR* was found to significantly reduce plasma cholesterol and non-HDL cholesterol levels in chow-fed animals at doses as low as 3×10^9^ genome copies/mouse. Whereas *Ldlr^−/−^Apobec1^−/−^* mice fed a western-type diet and injected with a control AAV8.null vector experienced a further 65% progression in atherosclerosis over 2 months compared with baseline mice, *Ldlr^−/−^Apobec1^−/−^* mice treated with AAV8.*mLDLR* realized an 87% regression of atherosclerotic lesions after 3 months compared to baseline mice. Immunohistochemical analyses revealed a substantial remodeling of atherosclerotic lesions.

**Conclusions/Significance:**

Collectively, the results presented herein suggest that AAV8-based gene therapy for FH may be feasible and support further development of this approach. The pre-clinical data from these studies will enable for the effective translation of gene therapy into the clinic for treatment of FH.

## Introduction

Familial hypercholesterolemia (FH) is a life-threatening genetic disease caused by mutations in the LDL receptor (*LDLR*) gene [1]. Patients with loss-of-function mutations in both *LDLR* alleles (homozygous FH – hoFH) develop atherosclerosis before age 20 and, if not treated, rarely survive past age 30. Patients with hoFH are minimally responsive to conventional LDL-lowering pharmacologic therapy. Orthotopic liver transplantation has been demonstrated to substantially reduce LDL-cholesterol (LDL-C) in hoFH patients, but obvious disadvantages and risks are associated with this approach [2], [3]. The current standard of care in hoFH is LDL apheresis, a physical method of purging the plasma of LDL-C which can transiently reduce LDL-C by more than 50% [4], [5], [6]. However, there is re-accumulation of LDL-C in plasma [7], and therefore apheresis has to be repeated every one to two weeks. Anecdotal evidence suggests that this procedure may delay the onset of atherosclerosis [8]; nonetheless it is laborious, expensive, and not readily available. Furthermore, although the procedure is generally well tolerated, the fact that it needs frequent repetition and IV access can be challenging for many patients and associated with morbidity. Therefore, there is a tremendous unmet medical need for new therapies for hoFH.

Liver-directed gene therapy has been tested as a possible alternative therapy for liver metabolic diseases such as hoFH. Initial attempts to treat FH with gene therapy utilized an *ex vivo* approach wherein autologous hepatocytes transduced with retroviruses containing *LDLR* cDNA were transplanted into homozygous FH patients [9]. Although this approach was well-tolerated by patients, the impact on cholesterol metabolism was modest and variable due in part to the limited amount of gene transfer achievable [9], [10]. More recently, attention has focused on the potential of liver directed *in vivo* gene therapy for hoFH.

Nearly all *in vivo* gene therapy based attempts to correct FH have used first generation adenoviral (Ad) vectors or helper-dependent adenoviral vector systems [11], [12], [13], [14]. While these vectors have proven to be quite efficient, they are associated with substantial toxicity due in part to capsid mediated activation of innate immunity and local and systemic inflammation [15], [16]. Compared to adenoviral constructs, vectors based on adeno-associated virus (AAV) demonstrate the attractive property of long-term expression without evidence of inflammation [15], [16], [17]. Initial AAV studies used AAV serotype 2 (AAV2) to express *LDLR* [17]. This approach led to a transient drop in the cholesterol of fat-fed *Ldlr^−/−^* mice but also triggered a transgene-specific immune response and loss of liver-associated vector DNA; furthermore transduction efficiency was low and efficacy was incomplete. AAVs based on novel capsids have recently been identified [18]; these vector candidates have shown impressive pre-clinical data. One particular vector -AAV8- has shown great promise in mouse and monkey models of liver directed gene transfer, including higher transduction efficiency, less pre-existing humoral immunity in humans, and diminished T cell responses to the capsid [19], [20], [21]. AAV mediated gene transfer with these new vectors showed long-term correction of the metabolic defect in fat-fed *Ldlr^−/−^* mice [22] and prevention of atherosclerosis in apoliporotein E deficient (*ApoE^−/−^*) mice [19] and fat-fed *Ldlr^−/−^* mice [22].

Intrinsic differences in the lipoprotein metabolism of FH animal models used in gene therapy studies and humans, however, limit the relevance of previous preclinical studies to the potential for human application. In humans, the liver synthesizes solely the full-length form of apolioprotein B (APOB), called APOB100, which contains in its carboxy terminal region the motif mediating binding to LDLR. However, mice express in liver high levels of the APOB mRNA editing catalytic polypeptide-1 (APOBEC1), which results in editing of the *ApoB* RNA transcript and the production of a truncated form of the APOB protein called APOB48, which does not bind to LDLR. Mice deleted in the *Apobec1* gene synthesize only APOB100 protein in the liver and thus more closely resemble human physiology. *Ldlr^−/−^Apobec1^−/−^* mice much more closely simulate the metabolic and clinical aspects of FH than do *Ldlr^−/−^* mice [23]. On a chow diet, these mice develop spontaneous and substantial hypercholesterolemia with a lipoprotein profile similar to that of human hoFH patients. Furthermore, in contrast to *Ldlr^−/−^* mice which require a high-fat diet to develop atherosclerosis and develop relatively simple macrophage-rich atherosclerotic lesions, *Ldlr^−/−^Apobec1^−/−^* mice fed a chow diet develop extensive atherosclerosis throughout the aorta, including most of the branch points; these lesions range in morphology from simple lesions of macrophage foam cells to more complex lesions containing smooth muscle cells and extracellular matrix [24]. Feeding a high-fat diet to *Ldlr^−/−^Apobec1^−/−^* mice further enhances the rate of progression of atherosclerosis [23]. To date, no gene therapy studies have evaluated FH correction in this mouse model.

For *in vivo* gene therapy of FH to become a clinical reality, pre-clinical studies need to be conducted using the proper combination of vector, transgene, and animal model that accurately reflects FH pathophysiology. The advent of AAV8 and the availability of *Ldlr^−/−^Apobec1^−/−^* mice provide an opportunity to critically evaluate the potential of gene therapy for hoFH using current models and gene transfer technology.

## Materials and Methods

### Animals

Male C57BL/6 *Ldlr^−/−^Apobec1^−/−^* mice were bred in house and have been described elsewhere [23]. For expression and efficacy studies, mice were given unrestricted access to water and were fed a standard chow diet. The vector was injected via an intravenous tail-vein injection with specified genome copies (gc)/mouse. Animals were sacrificed 35 days after gene transfer. For all studies, blood was obtained at least one times before and at designated time-points after gene transfer. For atherosclerosis regression studies, three groups of male mice (n = 10 per group) were given unrestricted access to water and were fed a high-fat western diet (0.15% cholesterol, 21% butterfat; DYETS, PA, USA) starting 8 weeks prior to vector injection and maintained on this diet throughout the experiment. After 8 weeks on the high-fat diet, prior to vector injection, one group of mice (baseline) was sacrificed for various analyses. The second and third groups were injected with 1×10^11^ GC of AAV8.TBG.*mLDLR* or AAV8.TBG.*nLacZ* vector, respectively. Eight weeks after vector treatment, mice were sacrificed for the indicated analyses. *Ldlr^−/−^Apobec1^−/−^ApoB^+/+^* mice were generated by crossing *Ldlr^−/−^Apobec1^−/−^* [23] with human *ApoB* transgenic mice [25]. *Ldlr^−/−^Apobec1^−/−^ApoB^+/+^* mice are viable, fertile, and do not display any gross physical or behavioral abnormalities. These animals were bred in house and maintained on a chow diet. All study protocols were approved by the Institutional Animal Care and Use Committee at The University of Pennsylvania (Protocol Approval #800791).

### Cloning and AAV Production

The mouse *Vldlr* and *Ldlr* AAV constructs were cloned in an AAV ITR flanked construct named pENN AAV TBG PI construct which drives expression from a thyroxine-binding globulin (TBG) hepatocyte specific promoter with a chimeric intron from Promega Corporation (Madison, Wisconsin) encoding the 5′-donor site from the first intron of the human b-globin and the branch and 3′-acceptor site from the intron located between the leader and body of an immunoglobulin gene heavy chain variable region. The cDNA sequences were amplified from PCR from constructs acquired from openbiosystems.com (*Ldlr*: genbank accession number BC019207; *Vldlr*: BC013622) with primers:

MLU-mLDLR F (gtaagcACGCGTaagctaaggatgagcaccgcggatctgat),

MLU-mLDLR R (TTGAttcACGCGTtcatgccacatcgtcctccaggctgaccatc),

MLU-mVLDLR F (gtaagcACGCGTgcgggcagcatgggcacgtccgcgcgctgg),

SAL-mVLDLR R (ggaatcGTCGACtcaagccagatcatcatctgtgcttacaac).

The *Vldlr* construct was generated by MluI-SalI insertion of the cDNA insert into pENN AAV TBG PI construct. *Ldlr* was cloned by MluI insertion of the cDNA amplicon into the same AAV construct. AAV vector was generated by triple transfection of the AAV ITR flanked construct with the packaging plasmid pAAV2/8 and the helper plasmid pdF6 as previously described [20], [21]. AAV vector particles were purified by double CsCl gradient banding as described in Wang et al [21].

### *En Face* Quantification of Atherosclerotic Lesions in the Aorta

Mice were anesthetized, the aorta was gently perfused with saline via the left ventricle, and the heart was cut off at the base and embedded in OCT. The rest of the aorta was removed and fixed in 10% formalin/PBS for at least 3 days. Aortic arch lesion area was quantitated using a method similar to that previously described [26], [27]. After the adventitial and adipose tissue was removed, the aortic arches were stained with oil red O solution (1.8% oil red O, wt/vol, in 60% isopropanol, filtered twice through a 0.2-µm filter) for 15 minutes and destained with 60% isopropanol for 5 minutes to eliminate background staining. The outer curvature of the arch was cut longitudinally, and the arch was laid open on a glass slide and mounted in Supermount (BioGenex). The image was captured with the use of a Leica MZIZ microscope and digitized, and the oil red O–stained lesion area was quantitated using the Image J image analysis system. All data capture and quantitation were performed in a blinded fashion.

### Immunohistochemistry and Quantification of Atherosclerosis Lesions in the Aortic Root

LDLR expression in liver was detected on frozen sections that were fixed in acetone (−20C) for 5 min, air dried, blocked with 1% goat serum in PBS for 20 min, and incubated for 1 h with a rabbit serum against LDLR (a gift from Dr. William Lagor and Dr. Gene C. Ness) diluted 1∶200 in blocking buffer [28]. After washing in PBS, the sections were treated for 30 min with FITC-labeled secondary antibodies against rabbit IgG diluted in blocking buffer (Vector Laboratories, 1∶200) and mounted with Vectashield containing DAPI (Vector). Fresh-frozen OCT-embedded hearts were used for Immunohistochemistry of lesions in the aortic root. Serial sections of the aortic root were mounted on masked slides. Sections were fixed in acetone, air-dried, and rehydrated in PBS containing 0.02% NaN_3_ and blocked with 1% BSA in PBS/NaN_3_ and 1% goat serum. For lesion analysis, sections were stained with oil-red-o or hematoxylin and eosin. Images were captured digitally with a video camera connected to a Leica microscope. The digitized images were analyzed with Image J image analysis software. Total lesion area was quantified by manual tracing of entire intimal lesion in 10 equally spaced aortic root sections per mouse. The acquisition of images and analysis of lesions were performed in a blinded fashion. For detection of macrophages, or VCAM-1, sections were reacted with rat antibodies against CD68 (clone FA-11, Serotec) and VCAM-1 (clone 429, BD Pharmingen) diluted 1∶200 and 1∶25 in blocking buffer, respectively. A Vectastain Elite ABC kit (Vector) was used according to the manufacturer's protocol to visualize bound rat antibodies. Collagen was detected by trichrome staining using a staining kit from Sigma following the manufacturer's protocol.

### Analytical Methods

The plasma total cholesterol levels were measured in individual mice at each time point with an enzymatic assay on a Cobas Fara II (Roche Diagnostic Systems Inc) with the use of Sigma reagents (Sigma Chemical Co). Plasma samples from groups of 5 mice were pooled at each time point and subjected to fast protein liquid chromatography (FPLC) gel filtration (Pharmacia LKB Biotechnology) on two Superose 6 columns. Cholesterol concentrations in the fractions were determined with an enzymatic assay (Wako Pure Chemical Industries, Ltd). For hepatic cholesterol and triglyceride analysis, liver was homogenized in a saline solution (∼250 mg liver/ml saline). Cholesterol and triglyceride were measured as described [29] after solubilization of lipid by deoxycholate using cholesterol reagent or triglyceride reagent and lipid lintrol standards.

### Statistical Analyses

Atherosclerotic lesion area data were subjected to a 1-way ANOVA. Experimental groups were compared with the baseline group by using the Dunnett test. Repeated-measures ANOVA was used to compare cholesterol levels among different groups of mice over time after gene transfer. Statistical significance for all comparisons was assigned at P<0.05. Graphs represent mean±SD values.

## Results

### *mLDLR* is more effective than *mVLDLR* in correcting hypercholesterolemia

Although *LDLR* is the logical transgene to use for gene therapy of FH, we and others have considered an alternative approach using the gene encoding the very-low-density lipoprotein receptor (*VLDLR*) which is a member of the *LDLR* super family that binds with high affinity to APOE containing lipoproteins such as VLDL [14], [17]. While this gene is not expressed in liver, vector mediated gene transfer of *VLDLR* to liver does partially ameliorate hypercholesterolemia in rabbits [13] and mice deficient in *Ldlr* [12], [13], [17]. One advantage of VLDLR is that it should be viewed as a self protein in hoFH patients and therefore should not elicit adaptive immune responses [12], [17].

**Figure 1:**
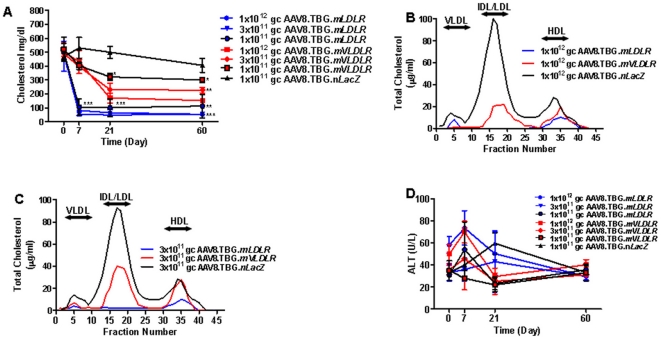
Evaluation of AAV8 encoding mouse *Vldlr* or mouse *Ldlr* in Ldlr-/-Apobec1-/- Mice. (A) Plasma cholesterol levels in *Ldlr-/-Apobec1-/-* mice after treatment with AAV8.TBG.*mVLDLR* or AAV8.TBG.*mLDLR* (n = 5 animals per dose group). Each point represents mean ± s.d. *P<0.05, **P<0.01, ***P<0.001 (B and C) Pooled mouse plasma from AAV-injected *Ldlr-/-Apobec1-/-* (n = 5) were analyzed by FPLC fractionation and the cholesterol content of each fraction was determined. (B) Lipoprotein profile of animals injected with 1×10∧12 GC of vector 28 days after treatment. (C) Lipoprotein profile of animals injected with 3×10∧11 GC of vector 28 days after treatment. (D) Plasma ALT levels in *Ldlr-/-Apobec1-/-* mice after treatment with AAV8.TBG.*mVLDLR* or AAV.TBG.*mLDLR* (n = 5 animals per dose group). Each point represents mean ± s.d. At all time points and doses examined, no significant differences in ALT were detected between AAV8.TBG.*mLDLR* and AAV8.TBG.*mVLDLR*.

A proximal step in the development of a clinical trial of gene therapy for hoFH is the selection of the transgene which, in the case of hoFH, could be *LDLR* or *VLDLR*. We performed a direct comparison of AAV8 mediated gene transfer of the murine versions of both genes at three doses (1×10^12^, 3×10^11^ and 1×10^11^ GC/mouse) in chow-fed *Ldlr^−/−^Apobec1^−/−^* mice using a liver specific promoter, thyroxine binding globulin (TBG). Within seven days of vector administration, a marked reduction in plasma cholesterol was realized in *Ldlr^−/−^Apobec1^−/−^* mice injected with AAV8.TBG.*mLDLR* at all doses compared to control AAV8.TBG.*nLacZ* treated animals, in which serum lipids were maintained at the high baseline levels throughout the study (Fig. 1A). In the low-dose (1×10^11^ GC/mouse) group, for example, plasma cholesterol levels significantly fell from a baseline level of 505±59 mg/dl to 112±34 mg/dl. AAV8.TBG.*mVLDLR* treated animals realized much less significant decreases in levels of total plasma cholesterol (Fig. 1A). We also examined FPLC fraction cholesterol levels 28 days after treatment with the high and (Fig. 1B) and intermediate-dose of vector (Fig. 1C). AAV8.TBG.*mLDLR* at both doses was considerably more effective than AAV8.TBG.*mVLDLR* in reducing levels of VLDL and IDL/LDL in *Ldlr^−/−^Apobec1^−/−^* mice; in contrast, a prominent IDL/LDL peak was detected at both doses of AAV8.TBG.*mVLDLR* treatment. No elevation of transaminases was detected in any group (Fig. 1D). Given these results, the remaining experiments focused exclusively on characterizing AAV8.TBG.*mLDLR* function *in vivo*.

### AAV8 mediated gene transfer of mLDLR is effective at low doses of vector

Critical to the success of gene therapy is the demonstration of efficacy at doses of vector that are safe and can be easily manufactured. Formal regulatory review of a proposed human study requires an assessment of the minimal dose that shows some metabolic/clinical effect [30]. Chow-fed male *Ldlr^−/−^Apobec1^−/−^* mice were treated with a single intravenous injection of AAV8.TBG.mLDLR; doses ranged from 1×10^12^ genome copies/mouse to 3×10^8^ genome copies (GC)/mouse. Thirty-five days after vector injection, mice were necropsied and livers were harvested and processed for presence and abundance of vector genomes and LDLR protein [21].

**Figure 2:**
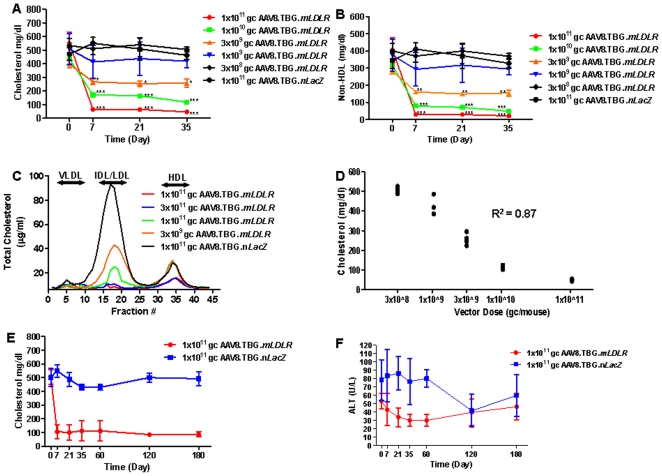
Evaluation of the minimum effective dose of AAV.TBG.*mLDLR* vector in *Ldlr-/-Apobec1-/-* Mice. Amounts of (A) Plasma cholesterol and (B) non-HDL cholesterol were evaluated in *Ldlr-/-Apobec1-/-* mice up to day 35 after treatment with different doses of AAV8.mLDLR (n = 9 animals per dose group). Each point represents mean ± s.d. *P<0.05, **P<0.01, ***P<0.001. (C) Pooled mouse plasma from AAV-injected *Ldlr-/-Apobec1-/-* (n = 5, per dose group) were analyzed by FPLC fractionation and the cholesterol content of each fraction was determined. (D) Dose response analysis of Day 60 samples examining cholesterol levels as a function of vector dose. (E) Plasma cholesterol and (F) Alanine transaminase were evaluated in *Ldlr-/-Apobec1-/-* mice up to day 180 days after treatment with 1×10∧11 GC of AAV8.TBG.*mLDLR* (n = 10) or 1×10∧11 GC of AAV8.TBG.*nLacZ* (n = 9). Each point represents mean ± s.d.

Correction of hypercholesterolemia was found to be dose dependent. With respect to total cholesterol (Fig. 2A) and non-HDL cholesterol (Fig. 2B), reduction was realized within seven days of vector treatment. The most significant decreases were observed at the highest treatment doses. Surprisingly, reduction was observed with doses as low as 3×10^9^ GC/mouse with total cholesterol levels dropping 35% from 397±26 mg/dl at baseline to 256±29 mg/dl on day 35 and non-HDL cholesterol levels decreasing by 45% from 284±19 mg/dl at baseline to 154±17 mg/dl (Fig. 2B) on day 35. This magnitude of correction is comparable to that seen in *Ldlr^−/−^Apobec1^−/−^* mice treated with 1×10^11^ GC/mouse of AAV8.TBG.*mVLDLR* (Fig. 1A), suggesting that AAV8.TBG.*mLDLR* is at least 30 fold more effective than AAV8.TBG.*mVLDLR*. With respect to FPLC fraction cholesterol levels, all doses of AAV8.TBG.*mLDLR*, except 3×10^8^ GC, led to substantial reductions in the IDL/LDL peak compared to AAV8.TBG.*nLacZ* treated *Ldlr^−/−^Apobec1^−/−^* mice. Only the higher doses of 1×10^11^ GC/mouse and 1×10^10^ GC/mouse also reduced the HDL-C levels compared to control *Ldlr^−/−^Apobec1^−/−^* mice (Fig. 2C). Regression analysis of the day 35 post-injection data revealed a clear dose-dependent response with regard to reduction of total cholesterol levels (Fig. 2D). Long-term studies revealed that treatment of *Ldlr^−/−^Apobec1^−/−^* mice with 1×10^11^ GC AAV8.TBG.*mLDLR* resulted in significant correction of cholesterol (Fig. 2E) in the absence of ALT elevation for up to 6 months after injection of vector (Fig. 2F). Collectively, these data reveal that 1×10^10^ GC/mouse is the minimum effective dose required for complete correction; furthermore, partial correction can be attained with as little as 3×10^9^ GC/mouse. Importantly, this correction was maintained for up to 6 months after treatment with no detectable inflammation.

**Figure 3:**
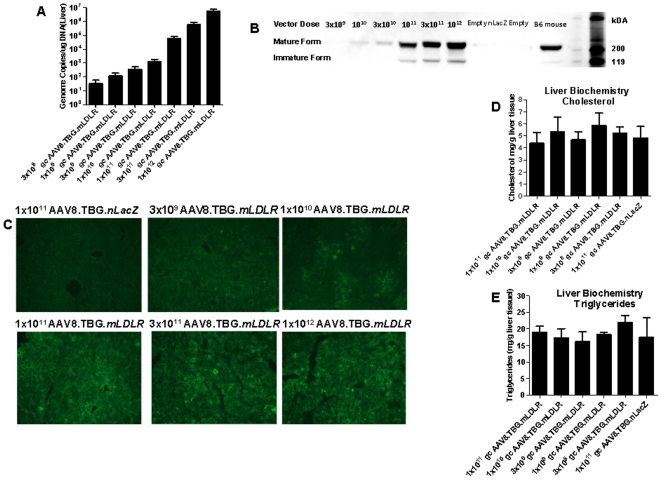
Efficacy of vector mediated transgene expression in liver. (A) Vector DNA expression in the liver. The amount of vector genomes in the liver *Ldlr-/-Apobec1-/-* mice (n = 9 mice per group) was assessed by Taqman analysis of total genomic DNA harvested at day 35 after vector administration. The limit of detection is 10 genome copies/µg DNA. (B) Membrane fraction of liver extract at day 35 was prepared from AAV-treated *Ldlr-/-Apobec1-/-* mice and subjected to Western blot analysis for the detection of transgene expression using LDLR specific antiserum. Positive control is liver extracts from B6 mice. Negative control is liver extracts from AAV8.TBG.*nLacZ* treated *Ldlr-/-Apobec1-/-* mice. (C) Histological analysis of mouse liver. Immunofluorescence staining was performed on mouse liver sections for detection of LDLR expression in *Ldlr-/-Apobec1-/-* mouse at day 35 after vector administration. Cryo-preserved liver section were prepared and probed with rabbit serum against LDLR followed by FITC labeled secondary antibody. Total levels of cholesterol (D) and (E) triglycerides were measured in the livers of *Ldlr-/-Apobec1-/-* mice at day 35. No significant differences were detected between any of the treatment groups (n = 5 mice per treatment group).

Taqman analysis revealed a dose dependent recovery of vector genomes from the liver (Fig. 3A). Using Western blot analysis, we detected LDLR immunoreactive protein in the liver of AAV8.TBG.mLDLR treated mice with vector doses equal to or greater than 1×10^10^ GC (Fig. 3B). At 1×10^10^ GC/mouse, only the mature form of the LDLR protein (160 kDa band) was detected, whereas in the higher dose groups, both the mature and immature incompletely glycosylated forms (120 kDa band) were present [31]. Immunofluroesence analysis revealed dose dependent expression of immunoreactive LDLR in liver (Fig. 3C); specifically, within 35 days of vector treatment, 60 to 70% of hepatocytes expressed immunoreactive LDLR following injection with 1×10^12^ GC/mouse whereas between 5 to 10% of hepatocytes expressed detectable LDLR at the lowest dose showing some metabolic improvement (i.e., 3×10^9^ GC/mouse). As expected, no LDLR protein was observed in the livers of AAV8.TBG.*nLacZ* treated *Ldlr^−/−^Apobec1^−/−^*mice (Fig. 3B and 3C). Importantly, over-expression of *mLDLR* did not result in the pathologic accumulation of hepatic cholesterol (Fig. 3D) or hepatic triglycerides (Fig. 3E) at any of the doses examined.

### AAV8.mLDLR Induces marked regression of atherosclerosis

**Figure 4:**
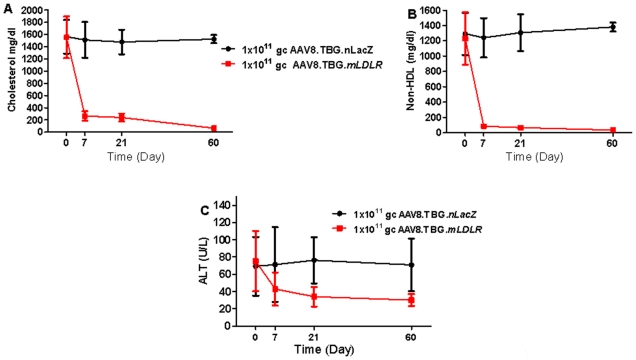
Evaluation of AAV8.TBG.*mLDLR* vector in high fat fed *Ldlr-/-Apobec1-/-* mice. Amounts of (A) Plasma cholesterol (B) non-HDL cholesterol, and (C) Alanine transaminase were evaluated in *Ldlr-/-Apobec1-/-* mice up to day 60 after treatment with 1x10∧11 GC of AAV8.TBG.*mLDLR* (n = 10) or 1×1011 GC of AAV8.TBG.*nLacZ* (n = 9). Each point represents mean ± s.d. *P<0.05, ‡ P<0.001.

Given that AAV8.TBG.*mLDLR* markedly lowered total cholesterol and non-HDL cholesterol, we next examined whether AAV8.TBG.*mLDLR* had any effect on the progression or even the regression of atherosclerotic lesions. Three groups of male *Ldlr^−/−^Apobec1^−/−^* mice were fed a high-fat western-type diet to hasten the progression of atherosclerosis. After two months of the atherogenic diet, one group of mice received a single intravenous injection of 1×10^11^ GC/mouse of control AAV8.TBG.*nLacZ* vector, one group received 1×10^11^ GC/mouse of AAV8.TBG.*mLDLR* vector, while a third baseline group of animals was necropsied for atherosclerosis lesion quantification. The mice who received vectors were maintained on the high-fat diet for an additional 60 days at which time they were necropsied. Animals that received the AAV8.TBG.*mLDLR* vector realized a rapid drop in total cholesterol from 1555±343 mg/dl at baseline to 266±78 at day 7 and to 67±13 by day 60 after treatment (Fig. 4A). By contrast, the plasma cholesterol levels of AAV8.TBG.*nLacZ* treated mice remained virtually unchanged from 1566±276 at baseline to 1527±67 when measured 60 days after vector (Fig. 4A). The same trend was observed with respect to non-HDL cholesterol (Fig. 4B). All animals developed slight increases in serum transaminanses following the 2 months on the high fat diet, which remained elevated following treatment with the AAV8.TBG.*nLacZ* vector but diminished three-fold to normal levels after treatment with the AAV8.TBG.*mLDLR* vector (Fig. 4C).

**Figure 5:**
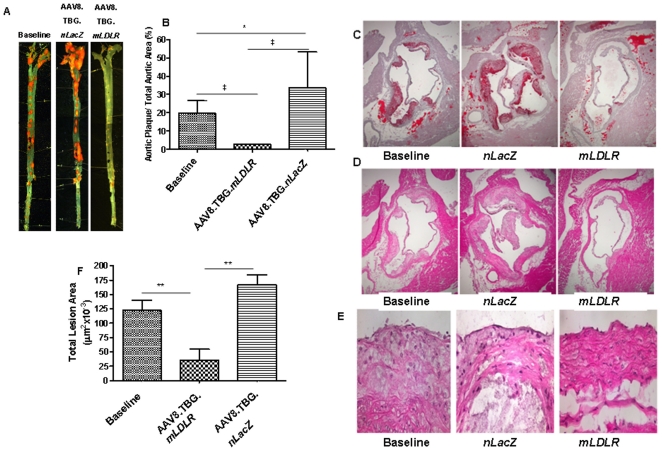
AAV8.TBG.*mLDLR* mediated regression of atherosclerotic lesions in high-fat fed *Ldlr-/-Apobec1-/-*mice. (A) En face Sudan IV staining. Mouse aortas were pinned and stained with Sudan IV, which stains neutral lipids. Representative aortas from animals treated with 1×10∧11 of AAV8.TBG.*nLacZ,* 1×10∧11 of AAV8.TBG.*mLDLR* at day 60 after vector administration (day 120 on high-fat diet), or at baseline (day 60 on high-fat diet) are shown. (B) The percent Sudan IV staining of the total aortic surface in baseline (n = 10), AAV.TBG.*nLacZ* (n = 9) and AAV8.TBG.*mLDLR* (n = 10) was determined as described under Materials and Methods. Aortic roots from these mice were stained with oil red o (C) or hematoxylin and eosin (H&E) (D) 10× magnification. Quantification was conducted on oil red o lesions (E) as described in the materials and methods. Each column represents mean ± s.d. *P<0.05, **P<0.01, ***P<0.001, ‡ P<0.001. (F) H&E stained aortic roots at 40× magnification show a thin fibrous cap and expanded necrotic core in lesions of baseline and AAV8.nLacZ treated mice compared to AAV8.mLDLR injected animals.

Evolution of pre-existing atherosclerotic lesions was assessed by two independent methods. In the first method the aortas were opened from the arch to the iliac bifurication and stained with Oil Red O (Fig. 5A); morphometric analyses quantified the percent of aorta stained with Oil Red O along the entire length of the aorta (Fig. 5B). Two months of high fat diet resulted in extensive atherosclerosis covering 20% of the aorta reflecting the baseline disease at the time of vector; this increased to 33% over an additional two month period following treatment with the AAV8.TBG.*nLacZ* vector, representing a 65% further progression in atherosclerosis. In contrast, treatment with the AAV8.TBG.*mLDLR* vector led to a regression of atherosclerosis by 87% over two months, from 20% of the aorta covered by atherosclerosis at baseline to only 2.6% of the aorta covered by atherosclerosis 60 days after vector administration.

In the second method, total lesion area was quantified in the aortic root (Fig. 5C–5F). This analysis revealed the same overall trends, with AAV8.TBG.*nLacZ* injected mice showing a 44% progression over 2 months compared to baseline mice, while AAV8.TBG.*mLDLR* injected mice demonstrating a 64% regression in lesion compared with baseline mice. In summary, expression of LDLR via injection of AAV8.TBG.*mLDLR* induced marked reduction in cholesterol and substantial regression of atherosclerosis over 2 months as assessed by 2 independent methods of quantification at 2 different sites within the aorta.

**Figure 6:**
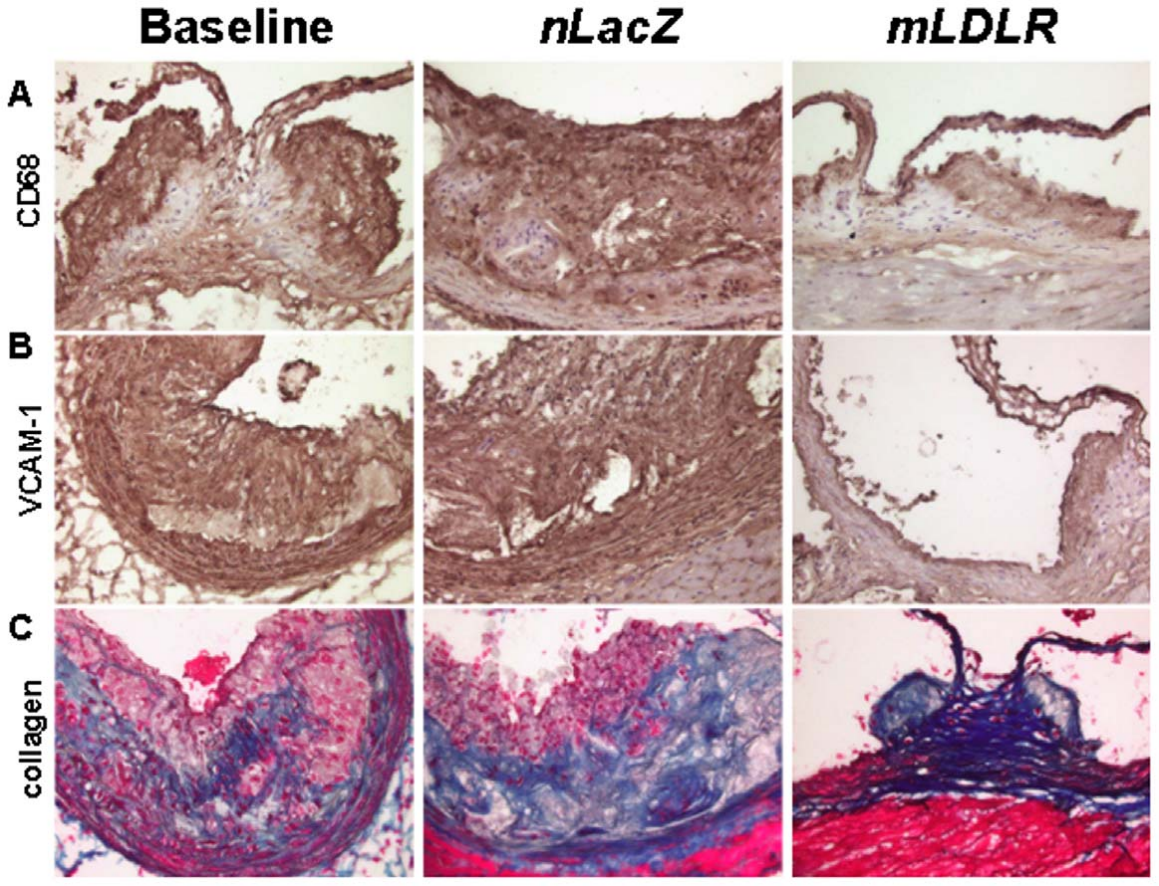
Immunohistochemical analysis of mouse atherosclerotic lesions. Representative aortic root sections immunostained for the foam cell marker CD68 (A), VCAM-1 (B), or Masson trichrome blue stain for collagen content (C). Original magnification, 40×. Note abundant immunostaining for foam cell marker, CD68 (brown), VCAM-1 adhesion molecules (also brown), and presence of collagen αblue) within lesion in baseline and AAV.TBG.*nLacZ* injected *Ldlr-/-Apobec1-/-*animals.

For further assessment of lesion remodeling, immunostaining was performed on fresh-frozen sections of the aortic root. Samples were examined for CD68, a foam cell marker (Fig. 6A), VCAM-1, an adhesion molecule that plays a role in atherosclerotic lesion formation (Fig. 6B), and collagen, a molecule that determines the mechanical stability of atherosclerotic plaques (Fig. 6C). Lesions in baseline treated groups were advanced, with many necrotic centers, CD68+ macrophages and macrophage-derived foam cells, and extensive VCAM-1 and collagen in the expanded neointima (Fig. 5F and 6A–6C) These results demonstrate the complexity of the lesions in the pre-injected aorta (i.e., that they had progressed past the early macrophage foam cell stage). AAV8.TBG.*nLacZ* treated animals displayed similar advanced pathology. In contrast, AAV8.TBG.*mLDLR* treated mice had much smaller lesions with decreased areas of CD68, VCAM-1, and collagen immunostaining in the intima and media. These results indicate a change in smooth muscle cell phenotype that is part of the wide-scale remodeling process that occurred during the 2 months in which systemic lipid levels where normalized.

## Discussion

Homozygous FH emerged as a model for liver-directed gene therapy in the 1980s with the demonstration of metabolic correction in patients following liver transplantation and genetic correction of deficient hepatocytes *ex vivo* with retroviruses[9], [10]. The optimal approach is *in vivo* targeting of hepatocytes following systemic delivery of vector, which is facilitated by the presence of fenestrae in liver capillaries[32]. Genotype/phenotype studies in patients with hoFH indicated the potential for efficacy in those with complete deficiency of *LDLR* following less than complete genetic reconstitution from gene therapy [9], [10], [33]. Finally, a simple measurement of serum lipids provides a specific, sensitive and relevant read out of gene transfer and clinical efficacy.

One of the biggest challenges in evaluating a novel gene therapy approach for the clinic is the identification of an authentic animal model for assessing efficacy and safety. This is particularly important when the underlying pathology of the disease may impact on vector performance as may be the case for the steatosis of liver in hoFH [34]. Animal models of hoFH have been described in rabbits and monkeys although they suffer from some significant limitations in terms of biology, genetics, available reagents and accessibility [35], [36], [37]. The mouse provides some substantial advantages especially as it relates to germ line engineering to render animal more “human like” as was the case with the mouse models used in this study.

Studies with AAV8 expressing *mLDLR* in the *Ldlr^−/−^Apobec1^−/−^* mouse showed impressive efficacy. Reductions in serum cholesterol of 35%, which according to human phenotype/genotype studies should be beneficial [33], were achieved with 3×10^9^ GC/mouse. This dose translates to 3×10^12^ GC for a 20 kg adolescent and 1×10^13^ GC for a 70 kg adult which is well within the reach of current manufacturing protocols [38]. Most importantly, we show that complete amelioration of hypercholesterolemia led to marked regression of atherosclerosis over two months despite continuation of the western-type diet. This result represents the fastest and most substantial regression of atherosclerosis seen in a murine model after reduction in plasma cholesterol.

A formal evaluation of safety awaits GLP toxicology studies, although our data to date demonstrated no toxicity up to 10^12^ GC. In fact, normalization of cholesterol following gene therapy actually improved the hepatotoxicity associated with steatosis that occurred when the animals were on a high fat diet (Fig. 4C). Recent reports suggest that the constitutive over-expression of *LDLR* may lead to pathological accumulation of lipids and cholesterol in hepatocytes and that the use of physiologically controlled expression elements is required for the design of FH gene therapy [39], . This effect has been seen *in vitro* and *in vivo* following delivery of *LDLR* expression plasmids under the control of strong viral promoters [39], [40]. No such pathologies (Fig. 3D–3E) were observed in our studies highlighting the importance of selecting the proper transgene/promoter/vector combination in designing gene therapy strategies.

Several additional aspects of AAV biology should be considered in possible clinical trials. We have shown that even low levels of pre-existing neutralizing antibodies (NAB) due to natural AAV infections will diminish gene transfer and potentially change vector bio-distribution[41]. AAV8 was isolated from a rhesus macaque and NABs to it are lower in humans than what is present to presumably human derived AAVs like AAV2 [18], [42]. However, some humans do show low levels of NAB to AAV8 [43]. Mouse studies like the ones described in this paper demonstrate the remarkable result of tolerance to antigenic transgenes [44] although our primate [45] and canine studies [20], [46] indicate that the threshold for T cell tolerance is lower in larger animals and presumably humans. Based on these findings we suggest that initial clinical trials be restricted to hoFH subjects who 1) are AAV8 NAB negative and 2) have at least one allele that expresses LDLR protein that spans most of its open reading frame to promote deletion of T cells to wild type LDLR. A final concern relates to the activation of T cells to capsids and killing of transduced hepatocytes which is a hypothesis that emerged from an AAV2 clinical trial in hemophilia [47], [48]. It seems prudent to consider a similar biology may occur with other AAV serotypes although our studies indicate this is unlikely to happen with AAV8 as it lacks the necessary dendritic cell interactions to effect cross presentation of capsids [49], [50].

In summary, our results suggest that the intravenous injection in mice of a low dose of an AAV8-based vector encoding the *LDLR* cDNA generates sustained expression of the LDLR protein in the liver without evidence of hepatic inflammation or toxicity and is highly effective in markedly reducing plasma cholesterol levels and regressing atherosclerosis in a “humanized” murine model of hoFH. These results suggest that AAV8-based gene therapy for hoFH may be feasible and support further development of this approach.
